# Correction: Kindlovits et al. Eight Weeks of Intermittent Exercise in Hypoxia, with or without a Low-Carbohydrate Diet, Improves Bone Mass and Functional and Physiological Capacity in Older Adults with Type 2 Diabetes. *Nutrients* 2024, *16*, 1624

**DOI:** 10.3390/nu16162681

**Published:** 2024-08-13

**Authors:** Raquel Kindlovits, Ana Catarina Sousa, João Luís Viana, Jaime Milheiro, Bruno M. P. M. Oliveira, Franklim Marques, Alejandro Santos, Vitor Hugo Teixeira

**Affiliations:** 1 Faculty of Nutrition and Food Sciences, University of Porto, FCNAUP, 4150-180 Porto, Portugal; nutri@raquelkin.com (R.K.); bmpmo@fcna.up.pt (B.M.P.M.O.); alejandrosantos@fcna.up.pt (A.S.); 2 Research Center in Sports Sciences, Health Sciences and Human Development, CIDESD, University of Maia, 4475-690 Maia, Portugal; acsousa@umaia.pt (A.C.S.); jlviana@umaia.pt (J.L.V.); 3 CMEP, Exercise Medical Centre Laboratory, 4150-044 Porto, Portugal; jaimemilheiro@yahoo.com; 4 Centre of Research, Education, Innovation and Intervention in Sport, CIFI2D, Faculty of Sport, University of Porto, 4200-540 Porto, Portugal; 5 Laboratory of Artificial Intelligence and Decision Support, Institute for Systems and Computer Engineering, Technology and Science (LIAAD, INESC-TEC), 4200-465 Porto, Portugal; 6 Laboratory of Biochemistry, Department of Biological Sciences, UCIBIO, REQUIMTE, Faculty of Pharmacy, University of Porto, 4050-313 Porto, Portugal; franklim@ff.up.pt; 7 Institute for Research and Innovation in Health, i3S, 4200-135 Porto, Portugal; 8 Research Center in Physical Activity, Health and Leisure, CIAFEL, Faculty of Sports, University of Porto, FADEUP, 4200-540 Porto, Portugal; 9 Laboratory for Integrative and Translational Research in Population Health, ITR, 4050-600 Porto, Portugal

In the original publication [1], there was a minor error in Figure 1 and Table 6. Unfortunately, Figure 1 presented a smaller text size than appropriate, making it difficult for the reader, in addition to the abbreviation “FiO2” instead of “FiO_2_”. Then, in Table 6, the basal lactate values between the groups were corrected and the lactate peak values were included.

The authors state that the scientific conclusions are unaffected. This correction was approved by the Academic Editor. The original publication has also been updated.

## Figures and Tables

**Figure 1:**
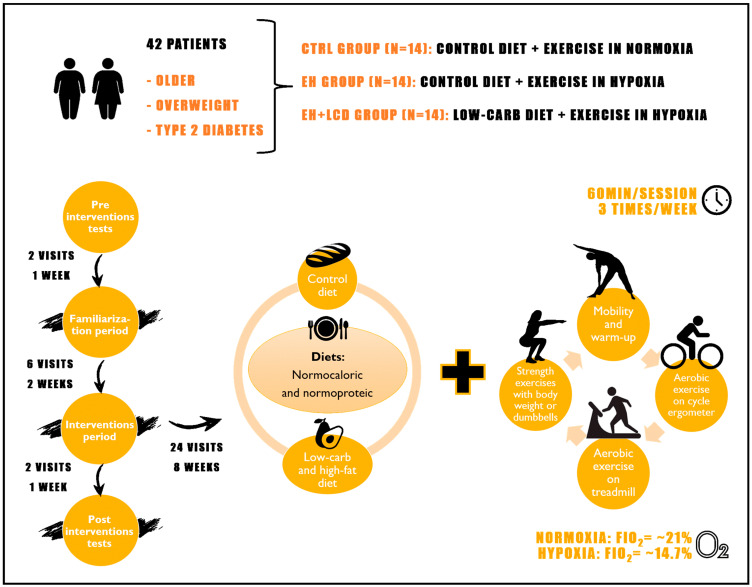
Illustrative scheme of the randomized clinical trial design.

**Table 6:** Functional and physiological capacity pre- and post-eight-week interventions in patients with T2DM.

Variables	CTRL Group			EH Group			EH + LCD Group			*p*-Value	
	Pre	Post	Δ	Pre	Post	Δ	Pre	Post	Δ	Moments	Groups
**Functional capacity**											
Maximum distance covered (m)	414.6 (62.0)	441.1 (70.0)	26.5 (49.1)	430.9 (98.8)	505.0 (87.7)	74.0 (46.0)	456.1 (70.9)	504.9 (101.0)	48.7 (58.9)	<0.001 *	0.030 #
Handgrip of dominant hand (kg)	23.5 (8.8)	24.7 (7.7)	1.2 (2.5)	23.5 (8.8)	26.9 (7.9)	3.4 (3.2)	23.0 (7.2)	25.5 (8.0)	2.4 (3.4)	<0.001 *	0.234
**Physiological capacity**											
Peak oxygen uptake (mL O_2_/min/kg)	21.4 (3.9)	24.5 (4.5)	3.8 (3.4)	21.8 (3.6)	29.2 (3.9)	10.0 (4.9)	19.6 (6.5)	27.7 (9.7)	7.4 (5.0)	<0.001 *	0.019 #
Basal heart rate (bpm)	77.6 (11.0)	73.7 (12.6)	6.1 (6.9)	75.2 (12.2)	70.8 (11.2)	3.8 (3.1)	73.5 (10.8)	72.8 (6.9)	0.4 (8.9)	0.020 *	0.134
Maximum heart rate (bpm)	127.8 (11.7)	121.8 (13.9)	8.0 (12.8)	149.1 (18.3)	132.5 (24.0)	13.5 (14.2)	129.2 (22.2)	132.0 (23.2)	2.6 (10.4)	0.008 *	0.023 #
Time to exhaustion (s)	344.6 (114.6)	489.0 (139.7)	155.0 (141.5)	536.2 (83.6)	629.5 (144.7)	124.5 (112.5)	460.4 (113.8)	684.1 (179.3)	217.5 (139.1)	<0.001 *	0.197
Peak workload (Watt)	178.4 (31.6)	248.1 (72.4)	76.5 (70.4)	232.8 (47.9)	283.7 (47.4)	54.1 (34.3)	230.2 (50.9)	249.4 (88.8)	32.7 (68.5)	<0.001 *	0.131
Peak workload (km/h)	4.6 (0.7)	5.8 (0.9)	1.3 (1.1)	5.7 (1.0)	6.6 (1.3)	0.9 (0.6)	5.6 (0.8)	6.5 (1.3)	1.1 (1.1)	<0.001 *	0.706
Basal lactate (mmol/L)	6.6 (1.9)	5.2 (1.8)	1.3 (1.3)	6.2 (2.5)	4.4 (2.6)	2.3 (1.5)	6.4 (2.2)	5.2 (3.2)	1.1 (2.3)	<0.001 *	0.183
Peak lactate (mmol/L)	9.3 (5.0)	6.1 (2.3)	3.2 (4.9)	8.6 (5.8)	6.8 (3.5)	2.2 (2.9)	7.9 (3.7)	6.5 (4.4)	1.4 (3.0)	0.001 *	0.690

Data are presented as the mean (standard deviation). The post hoc Tukey test was used to assess differences between groups. *p*-values represent a two-way repeated-measures ANOVA; “moments” compare the overall mean for the pre- and post-evaluations; and “groups” is the interaction term comparing the time variations among the groups. * = significant differences between results pre- and post-eight-week intervention. #: Maximum distance covered (m): the EH group increased more than the EH + LCD and CTRL groups; peak oxygen uptake (mL O_2_/min/kg): the EH and EH + LCD groups increased more than the CTRL group; maximum heart rate (bpm): the EH + LCD group increased more than the CTRL and EH groups. Δ = Changes from baseline to the eighth week.
